# Avoidant/Restrictive Food Intake Disorder Is Common in Adult and Pediatric Patients with Celiac Disease and Non-Celiac Gluten Sensitivity

**DOI:** 10.3390/nu18101585

**Published:** 2026-05-16

**Authors:** Micaela Atkins, Blythe Peterson, Kyle Staller, Braden Kuo, Bethlehem Michael, Margaret Savage, Maureen M. Leonard, Helen Burton-Murray

**Affiliations:** 1 Division of Pediatric Gastroenterology, Massachusetts General Hospital, Boston, MA 02114, USA; 2 Harvard Medical School, Boston, MA 02115, USA; 3 Division of Gastroenterology, Massachusetts General Hospital, Boston, MA 02114, USA; 4 Division of Gastroenterology, Department of Medicine, Columbia University Irving Medical Center, New York, NY 10032, USA; 5 David Geffen School of Medicine, UCLA, Los Angeles, CA 90095, USA; 6 Dr. Kiran C. Patel College of Allopathic Medicine, Nova Southeastern University, Fort Lauderdale, FL 33328, USA

**Keywords:** celiac disease, non-celiac gluten sensitivity, avoidant/restrictive food intake disorder, disordered eating

## Abstract

**Background/Objectives**: Avoidant/restrictive food intake disorder (ARFID), a non-body image-based eating disorder, has been described in several chronic gastrointestinal diseases, but little is known about ARFID in celiac disease (CeD) and non-celiac gluten sensitivity (NCGS). We aimed to identify the prevalence and characteristics of ARFID among adult and pediatric patients presenting for consultation of gluten-related disorders longitudinally. **Methods:** We reviewed 386 consecutive referrals (ages 2–82 y; 68% female) to a tertiary care celiac center from June 2019 to June 2022. Eligible patients had histologically confirmed CeD or were classified as NCGS based on gluten-related symptoms but negative serologies and histology. Masked coders applied *DSM-5* criteria for ARFID at initial presentation and one-year follow-up visit. **Results:** Out of 386 patients, 253 had biopsy information and were included in the study. ARFID symptoms were present in 19% (47/253) of patients at consultation and in 17% (22/126) at follow-up. ARFID symptoms were equally present among patients with CeD and NCGS and were not associated with the presence or duration of a gluten-free diet, but patients with ARFID were more likely to be on a non-gluten-free diet at presentation. In multivariate analysis, the likelihood of having ARFID symptoms at consultation increased with a history of weight loss (OR 2.87, 95% CI 1.36–6.30). **Conclusions:** We found that almost one-fifth of patients with CeD and NCGS had symptoms of ARFID at consultation. At follow-up, among a smaller cohort, ARFID prevalence remained similar, although some patients no longer had ARFID symptoms, and some had new ARFID symptoms develop. Further longitudinal research is needed to understand the risk and maintenance factors of ARFID in the context of CeD and NCGS.

## 1. Introduction

Celiac disease (CeD) is an immune-mediated multisystemic disorder driven by gluten ingestion [1]. Adherence to a lifelong gluten-free diet (GFD) is the only available treatment for CeD, and to be effective, GFD requires patients with CeD to diligently monitor what they eat. The GFD is also the primary treatment for non-celiac gluten sensitivity (NCGS), a syndrome characterized by gluten-related symptoms in the absence of CeD or wheat allergy [2].

While the prevalence of eating disorders is unknown in patients with NCGS, eating disorders in CeD are common and occur more frequently than in the general population [3,4,5]. This may be related to a genetic predisposition; one study showed several genes associated with anorexia nervosa are differently expressed in children with active CeD compared with controls [6]. However, there is growing concern that the focus on food and dietary restrictions inherent to gluten-related disorders may put some patients at risk for disordered eating [7,8,9].

Avoidant/restrictive food intake disorder (ARFID) is an eating disorder in which restrictive eating from insufficient volume or variety of food results in health or psychosocial consequences [10]. Restrictive eating in ARFID is not driven by concerns about shape or weight, but instead motivated by one or more of the following currently recognized presentations: sensitivity to sensory characteristics of food, lack of interest in eating, or fear of aversive consequences of eating [10]. Restrictive eating in ARFID cannot be otherwise explained by a medical condition [10], which makes diagnosing this disorder in the context of chronic gastrointestinal (GI) conditions challenging [11].

Recent cross-sectional studies have found a high prevalence of ARFID symptoms in adults with CeD; with 57% of patients screening positively in one study using the ARFID symptom checklist [12] and 49% in another using the Nine Item ARFID Screen [13]. However, these self-report tools are not validated in GI populations and do not apply the *Diagnostic and Statistical Manual for Mental Disorders (DSM-5)* ARFID criteria [10], which requires clinical judgment. Although there is growing interest in the intersection between gluten-related disorders and disordered eating [9,14], factors that put patients at risk for ARFID in the context of CeD remain poorly understood, with even less evidence in NCGS. In both disorders, some patients may become excessively worried about the risk of gluten ingestion, resulting in generalized dietary restrictions and ultimately ARFID, but this is yet to be explored.

In this retrospective study, we applied *DSM-5* ARFID criteria [10] to identify the prevalence and associated nutritional status-related characteristics of ARFID among pediatric and adult patients presenting for consultation of gluten-related disorders at initial presentation and at one-year follow-up.

## 2. Materials and Methods

Consecutively referred patients (*N* = 386, ages 2–82, 68% female) who presented for evaluation for gluten-related complaints to a tertiary care celiac center from 1 June 2019 to 1 June 2022 were identified under an IRB-approved protocol. Two masked coders (BM and MS) reviewed medical records beginning at consultation and including subsequent notes in the following year. Demographics, presence of GI- and non-GI symptoms, comorbidities, and CeD information were extracted.

Coders were trained by a pediatric gastroenterologist (MA) and a clinical psychologist (HBM) to systematically review medical records for evidence of any eating disorder symptoms, including restrictive eating, and using clinical judgement, completed an established *DSM-5* diagnostic checklist [15,16,17] to indicate the presence or absence of criteria for ARFID, anorexia nervosa, bulimia nervosa, binge-eating disorder, and other specified eating disorders. Coders conferred “Definite ARFID” when cases met all *DSM-5* ARFID criteria. Coders conferred “Potential ARFID” when cases met some criteria for ARFID, but not enough information was available to make a full diagnosis; for example, one case had an extremely limited diet (e.g., 5 foods), but there was a lack of documentation about psychosocial and/or medical impairments related to diet. The “Definite ARFID” and “Potential ARFID” cases were combined and categorized as “ARFID symptoms.” Coders met on a weekly basis with a pediatric gastroenterologist (MA) to discuss ambiguous cases, which were adjudicated by a psychologist (HBM).

**Figure 1:**
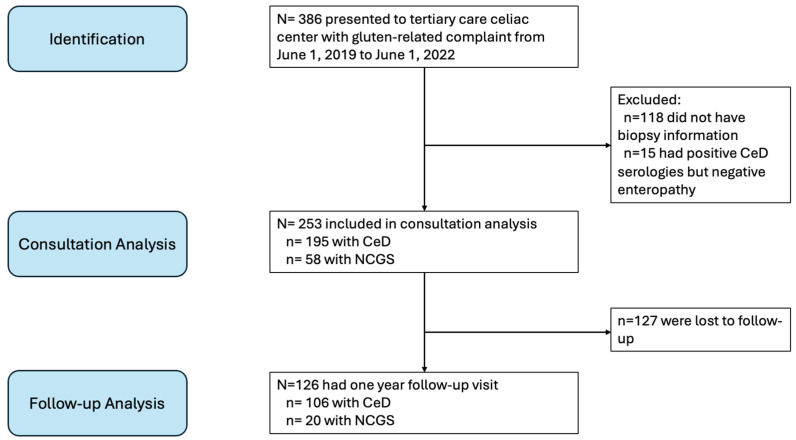
Patients included in consultation analysis and follow-up analysis; CeD = celiac disease; NCGS = non-celiac gluten sensitivity.

CeD was operationalized by enteropathy reported as Marsh 3, a standardized histologic marker of CeD activity [1]. Patients were classified as NCGS if they had gluten-related symptoms but negative serologies (tissue transglutaminase IgA, anti-endomysial IgA, and/or deaminated gliadin IgG) and no enteropathy on endoscopic biopsy. For purposes of comparison, we excluded patients with unclear CeD status from our analysis (i.e., patients without documented biopsies or those with positive serologies but no enteropathy, see Figure 1).

We also extracted nutritional information, including dietary history and weight/growth patterns for all patients from physician or dietitian notes. Patients were defined as having weight loss/poor weight gain or poor linear growth based on note documentation. Non-GFD exclusion diets were classified as any diet that eliminated specific foods or food groups beyond gluten (e.g., a dairy-free diet). For patients who had one-year follow-up visits, we also extracted dietary history, including time on the GFD and presence of non-GFD diets, as well as GI and non-GI symptoms. Patients were classified as having “active celiac disease” at the follow-up visit if there was documentation in a physician’s note about disease activity, including elevated serologies or positive histology within the past six months.

We calculated the prevalence of cases classified as “ARFID symptoms” (Definite + Potential ARFID) at consultation and one-year follow-up—among the whole cohort of patients presenting for consultation and separately among those with clear CeD status (CeD or NCGS). Among those with clear CeD status, we conducted Mann–Whitney U tests for continuous and Chi-square or Fisher’s exact test for categorical data, comparing those with and without ARFID symptoms. A sensitivity analysis was performed in the pediatric and adult cohorts. To determine effect size, we calculated Pearson’s *r* for continuous and Cramér’s *V* for categorical variables (0.1 = small, 0.3 = medium, 0.5 = large). Multivariate logistic regression was performed with predictors selected based on nutritional status-related factors identified in a univariate screen.

## 3. Results

Of the 386 patients who presented for consultation, ARFID symptoms were present in 69 patients (18%, 69/386), with 25 meeting criteria for “Definite ARFID” and 44 meeting criteria for “Potential ARFID”. For our analyzable sample, 133 patients were excluded because of unclear CeD status (118 with no histology information, and 15 with positive CeD serologies, but no enteropathy)—leaving 253 (ages 2–79, 73.5% female) patients with CeD or NCGS included in analysis (see Figure 1).

### 3.1. ARFID Symptoms at Consultation Visit

**Figure 2:**
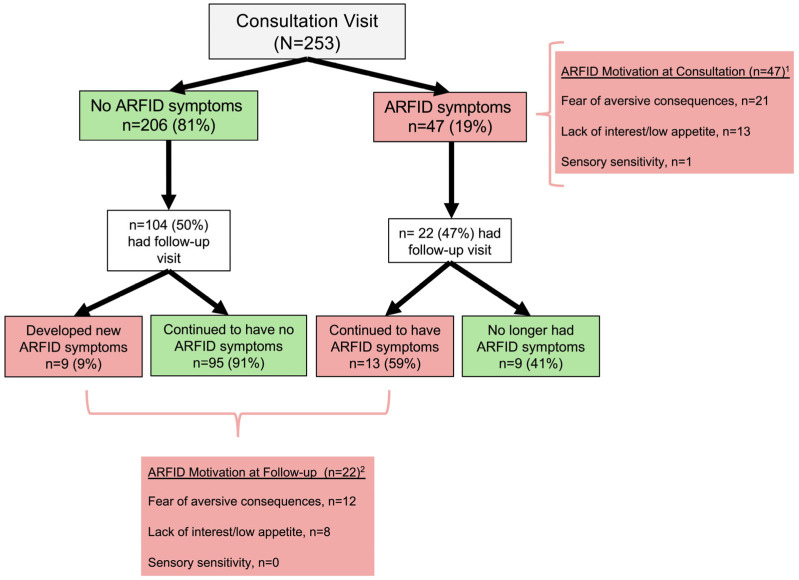
Note. ARFID = avoidant/restrictive food intake disorder. ^1^ Determined for 35/47 patients. ^2^ Determined for 20/22 patients.

Among the 253 patients with clear CeD status, ARFID symptoms were present in 19% (*n* = 47; 19 “Definite ARFID”, 28 “Potential ARFID”) at consultation, with an equal prevalence in those with CeD (18%, 36/195) and NCGS (19%, 11/58). The primary motivation for restrictive eating was most commonly fear of aversive consequences of eating (in 60%), which was followed by lack of appetite (in 37%). Only one patient had sensory sensitivity as the primary motivation for restrictive eating (see Figure 2).

Univariate comparisons showed that patients with ARFID symptoms were more likely to have a history of weight loss/poor weight gain (72.3% vs. 37.7%, small-medium effect) and be following a non-GFD exclusion diet (42.6% vs. 26.3%, small effect) (see Table 1). Patients with ARFID symptoms were also more likely to have a history of developmental disorder (21.3% vs. 8.3%, small effect). There was no difference in age, sex, GI symptom presence, or psychiatric comorbidities (Table 1).

Multivariate analysis of nutritional status-related factors showed that the odds of having ARFID symptoms at consultation increased with decreasing BMI (OR = 0.90, 95%CI = 0.81–0.98) and with a history of weight loss or poor weight gain (OR = 2.87, 95%CI = 1.36–6.30). A history of non-GFD exclusion diet use was not a significant predictor (Table 2).

Sensitivity analyses exploring the pediatric and adult cohorts alone at consultation showed that ARFID symptoms were present in 23% (28/123) of pediatric patients and in 15% (19/130) of adult patients. In both cohorts, patients with ARFID symptoms were more likely to have a lower BMI percentile/BMI and a history of weight loss/poor weight gain (see Supplemental Tables S1 and S2). Both cohorts also had higher percentages of patients with ARFID symptoms on a non-GFD exclusion diet in addition to the GFD compared to those without ARFID symptoms; however, this was only statistically significant in the pediatric group (39.2% vs. 18.1%, small effect, Supplemental Table S1).

### 3.2. ARFID Symptoms at One-Year Follow-Up Visit

Of the 253 patients who were included in the analysis at consultation, 50% (126/253) had a one-year follow-up visit. In this group, 17% (22/126) met criteria for ARFID symptoms at the follow-up visit (10 with “Definite ARFID” and 12 with “Potential ARFID”). Of the 22 patients with ARFID symptoms at follow-up, 13 (59%, 13/22) patients had also met criteria for ARFID symptoms at consultation, and 9 patients (41%, 9/22) had developed new ARFID symptoms between initial consultation and one-year follow-up visit (see Figure 2). The primary motivation for ARFID symptoms at the one-year follow-up was most commonly fear of aversive consequences (in 60%), followed by lack of interest/low appetite (in 40%).

Patients with ARFID symptoms at follow-up were more likely to have interval weight loss/poor weight gain (59.1% vs. 8.7%, large effect) and, in the pediatric population, interval poor linear growth (33.3% vs. 5.5%, medium-large effect). Dietary factors (i.e., time on the GFD, use of non-GFD exclusion diet) did not significantly differ between groups, similar to CeD activity and presence of GI symptoms, but patients with ARFID symptoms were more likely to report the presence of non-GI symptoms (e.g., headaches, brain fog, and fatigue) (68.2% vs. 41.7%, small effect). Patients with and without ARFID symptoms reported the presence of symptoms from gluten ingestion at similar rates (see Table 3). In logistic regression analysis, an interval history of weight loss/poor weight gain was associated with increased odds of ARFID symptoms at follow-up (OR = 18.57, 95% CI 5.90–66.64) (see Table 4).

## 4. Discussion

In this retrospective study of patients with CeD and NCGS, ARFID symptoms were common: present in 19% of patients at consultation and 17% at follow-up. Our findings add to the literature on disordered eating in gluten-related disorders, and to our knowledge are the first to report the prevalence of ARFID symptoms in NCGS. Importantly, we identified candidate factors associated with ARFID, notably compromised weight status (both low BMI and weight loss/poor weight gain) at consultation and interim weight loss/poor weight gain by one-year follow-up.

We found a lower ARFID symptom prevalence than what has been previously reported using ARFID self-screening tools in patients with self-reported CeD (49% using the Nine Item ARFID screen [13]) and biopsy-confirmed CeD (57% using the ARFID-checklist [12]). Self-report tools may over-inflate ARFID rates in chronic GI populations [13], as the cutoffs used to date may not be specific enough to separate normative, appropriate caution in dietary adherence from problematic restriction [18]. Our findings are based on a stricter classification framework, which involved a detailed review of clinician notes to discriminate between adaptive versus pathological restriction [18]. Although lower, our prevalence estimate is no less clinically significant, given the well-established associations of restrictive eating in CeD with lower quality of life scores [19,20] and nutritional consequences [9].

Similar to prior studies of ARFID in CeD [12] and in other chronic GI diseases [21], fear of aversive consequences of eating was the primary driver of restriction in our cohort of patients with ARFID symptoms. Patients with gluten-related disorders should exercise some degree of caution about the consequences of eating to maintain dietary adherence [18]. Survey studies in adults and qualitative work in children with CeD have demonstrated high rates of food neophobia [22] and hypervigilance with heightened anxiety of gluten cross-contact [14]. However, previous work in CeD has not shown that increased restriction is associated with improved GFD adherence [12,19]. In our study, patients with and without ARFID symptoms had similar frequencies of perceived gluten-related symptoms, which suggests that the significant food avoidance in our sample was not necessarily, or only, related to an elevated fear of gluten ingestion. 

We also did not find a relationship between the presence of ARFID symptoms and GI symptoms, which is in line with other studies on restrictive eating in CeD [12,19]. At follow-up, those with ARFID symptoms had increased rates of extraintestinal symptoms with a small effect. Extraintestinal symptoms (e.g., fatigue, headache, and brain fog) have been well described in both NCGS [23,24] and CeD [25], and are present in over half of patients [24,26]. As they may persist despite the GFD [26], it is possible that lingering symptoms prompt further food restriction.

Research in other chronic GI diseases has questioned whether ARFID symptoms may be a marker of GI disease activity or severity rather than a disorder itself, but our study suggests they are not. In research on inflammatory bowel disease, the prevalence of self-reported ARFID symptoms is higher in patients with active disease, with one study reporting only 11% of adults with quiescent ulcerative colitis screening positive on an ARFID self-report survey [27]. Active mucosal inflammation does not appear to be driving restrictive eating in CeD, as ours and prior studies [12] show no difference in disease activity between those with and without ARFID symptoms. In the same vein, we found no difference in ARFID symptoms between patients with CeD versus NCGS. Assessing restrictions in NCGS is particularly challenging as NCGS can be controversial and poorly understood. Patients with NCGS may be more likely to be diagnosed with disordered eating [18] because it is difficult to assess if restrictions are beyond what would be expected in the absence of objective biomarkers of disease activity. Prior research has shown the presence of a nocebo effect with NCGS [28,29], and it is possible that expectations around potential gluten exposure may prompt GI symptoms and then increased dietary restrictions. In both NCGS and CeD, the presence of persistent symptoms may instead reflect a comorbid disorder of gut–brain interaction (DGBI), such as irritable bowel syndrome or functional dyspepsia. There is evidence for overlap of DGBIs with both NCGS [30] and CeD [31], and the association between DGBIs and ARFID is well-established [32].

Patients with ARFID in our sample were more likely to follow a non-GFD exclusion diet at consult (albeit not significant on multivariate analysis), and a higher percentage (although not statistically significant) group followed a non-GFD exclusion diet at follow-up. A recent study found that over half of adults with biopsy-confirmed CeD reported avoiding additional foods beyond the GFD [19]. Our study found lower, but still notable, rates of non-GFD exclusion diets—in 29% of patients at consult and 24% of patients at follow-up. As patients with additional food avoidance have lower quality of life scores and increased anxiety and depression scores [19], providers should consider taking dietary histories that screen for dietary exclusions beyond gluten.

Importantly, we found that having a history of weight loss/poor weight gain was associated with an almost three times higher likelihood of having ARFID symptoms at consult, mirroring findings in neurogastroenterology samples [16,21], and if this was present at follow-up, patients were over 18 times more likely to have ARFID symptoms. This suggests that ARFID evaluation may be particularly relevant in patients presenting with weight/growth difficulties.

This study has several limitations related to its retrospective design, and specifically, reliance on free-text documentation in notes. This retrospective method of ARFID classification is subject to observer error and may under- or overestimate the true prevalence. Of the cases of ARFID symptoms identified in this study, more than half (44 at consultation and 12 at follow-up) did not have adequate chart information to fully confer a diagnosis of ARFID. It is unlikely that clinical notes fully capture patients’ eating behaviors, including motivations and functional impairments, and further research is certainly needed to corroborate findings with ARFID clinician diagnostic interviews. Our classification of NCGS also has limitations, as it was based on the presence of symptoms without positive serology or enteropathy, but did not involve a blinded gluten challenge. Finally, only half of our cohort had a one-year follow-up visit, so it is likely that we will have an incomplete view of this relationship over time. 

## 5. Conclusions

Our study identified ARFID symptom presence in approximately one in five patients with gluten-related disorders at two different time points, and screening for restrictive eating in this population is warranted. Of the patients with ARFID symptoms at the follow-up visit, over half had ARFID symptoms at consultation, suggesting that this disorder may persist longitudinally without intervention. Of equal importance, we also found that 40% of patients with ARFID symptoms at follow-up had developed symptoms since consultation, which highlights a need for a better understanding of how to prevent ARFID development in vulnerable populations. We argue that assessing the patients’ relationship with food and eating is as important as assessing comorbid GI and extraintestinal disorders in patients with gluten-related disorders. Guidance on the GFD should include a discussion of the psychological impact, beyond just assessing compliance, and clinicians should exercise notable caution with those presenting with weight loss/poor weight gain, or those on additional non-GFD exclusion diets. Future prospective studies are needed to better understand which factors put patients with gluten-related disorders at risk for ARFID symptoms. 

## Figures and Tables

**Table 1:** Characteristics of patients by ARFID symptom presence at CeD consultation (*N* = 253).

Consultation	ARFID Symptoms (*n* = 47)	No ARFID Symptoms (*n* = 206)	*p*-Value ^1^	V or *r* ^2^
Age at consult, median years (IQR)	15 (10–25.5)	18 (13–39.8)	0.08	0.12
Pediatric patients, age < 18 years, *n* (%)	28 (59.6%)	95 (46.1%)	0.13	0.10
Sex–female, *n* (%)	31 (66.0%)	155 (75.2%)	0.26	0.08
Race, *n* (%)			0.34	0.15
American Indian or Alaskan Native	1 (2.13%)	0 (0%)		
Asian	0 (0%)	1 (0.5%)		
Black or African American	1 (2.13%)	2 (1.0%)		
Native Hawaiian or Pacific Islander	0 (0%)	0 (0%)		
White	38 (80.9%)	180 (87.4%)		
Other	3 (6.4%)	10 (4.9%)		
Unknown	4 (8.5%)	13 (6.3%)		
Ethnicity, *n* (%)			0.61	0.06
Hispanic/Latino(a)	4 (8.5%)	10 (4.9%)		
Not Hispanic/Latino(a)	40 (85.1%)	183 (88.8%)		
Unknown	3 (6.4%)	13 (6.3%)		
BMI, median (IQR)				
BMI, median (IQR)—patients ≥ 18 years	18(20.2–23.4)	24.4(21.6–28.2)	<0.01	0.26
BMI percentile, median (IQR)—patients < 18 years	24.2(7.32–68.7)	58.1(31.2–78.4)	<0.01	0.28
Biopsy-confirmed celiac disease, *n* (%)	36 (76.6%)	159 (77.2%)	1	0.01
On GFD at consultation, *n* (%)	34 (72.3%)	132 (64.1%)	0.37	0.07
On non-GFD exclusion diet at consultation, *n* (%)	20 (42.6%)	54 (26.3%)	0.04	0.14
Presence of GI Symptoms at consult, *n* (%) ^3^	47 (100%)	192 (93.2%)	0.14	0.12
Presence of non-GI symptoms at consult, *n* (%) ^4^	44 (93.6%)	162 (84.4%)	0.16	0.11
History of weight loss/poor weight gain, *n* (%)	34 (72.3%)	77 (37.7%)	<0.01	0.27
History of poor linear growth in pediatric patients, *n* (%)	13 (27.7%)	24 (11.8%)	0.01	0.17
Comorbidities, *n* (%)				
History of anxiety	15 (31.9%)	55 (26.8%)	0.60	0.04
History of depression	10 (21.3%)	32 (15.6%)	0.50	0.06
History of other psychiatric disorder	3 (6.38%)	12 (5.85%)	1	<0.01
History of developmental disorder ^5^	10 (21.3%)	17 (8.33%)	0.02	0.16

Note: ARFID = avoidant/restrictive food intake disorder, BMI = body mass index, GFD = gluten-free diet, GI = gastrointestinal. ^1^ Continuous variables were analyzed with Mann–Whitney U tests; categorical variables were analyzed with chi-square or Fisher’s exact tests when cell counts <5. ^2^ Effect sizes were calculated using Pearson’s *r* for continuous variables and Cramér’s V for categorical variables. ^3^ GI symptoms included: abdominal pain, diarrhea, bloating, constipation, nausea, vomiting, increased flatulence, dysphagia, regurgitation, belching, heartburn/reflux, and unspecified bowel changes. ^4^ Non-GI symptoms reported included: headaches, brain fog, fatigue, joint pain, rash, and dizziness. ^5^ Developmental disorders included: developmental delay (speech, expressive language, gross motor), learning disability, and tic disorder.

**Table 2:** Logistic regression analysis for predictors of ARFID symptom presence at consult (*N* = 253).

	OR (95%CI)	*p*-Value
Age	1.0 (0.97–1.02)	0.80
Sex	0.93 (0.44–2.03)	0.86
BMI at consult	0.90 (0.81–0.98)	0.02
On a non-GFD exclusion diet	1.85 (0.88–3.88)	0.10
History of weight loss/poor weight gain	2.87 (1.36–6.30)	<0.01

Note: Age and sex are included as clinically relevant covariates; GFD = gluten-free diet. BMI = body mass index.

**Table 3:** Characteristics of patients by ARFID symptom presence at CeD follow-up visit (*n* = 126).

One-Year Follow-Up	ARFID Symptoms (*n* = 22)	No ARFID Symptoms (*n* = 104)	*p*-Value ^1^	V or *r* ^2^
On GFD at follow-up, *n* (%)	19 (86.4%)	97 (93.3%)	0.51	0.10
Time on GFD, median months (IQR)	20 (13.5–37)	16 (11–54)	0.18	0.13
On non-GFD exclusion diet at follow-up, *n* (%)	7 (31.8%)	23 (22.1%)	0.57	0.09
Active celiac disease at follow-up, *n* (%) ^3^	3 (13.6%)	17 (16.3%)	1	0.03
Interval weight loss/poor weight gain, *n* (%)	13 (59.1%)	9 (8.7%)	<0.01	0.50
Interval poor linear growth in pediatric patients, *n* (%)	5 (33.3%)	3 (5.5%)	<0.01	0.43
Presence of GI symptoms at follow-up, *n* (%) ^4^	14 (63.6%)	50 (48.1%)	0.28	0.12
Presence of non-GI symptoms at follow-up, *n* (%) ^5^	15 (68.2%)	43 (41.7%)	0.04	0.2
GI symptoms improved at follow-up, *n* (%)	16 (72.7%)	87 (83.7%)	0.37	0.12
Presence of patient-reported gluten-related symptoms, *n* (%)	12 (70.6%)	75 (80.6%)	0.54	0.09

Note: ARFID = avoidant/restrictive food intake disorder, GFD = gluten-free diet, GI = gastrointestinal. IQR = interquartile range, Q1–Q3. ^1^ Continuous variables were analyzed with Mann–Whitney U tests, categorical variables were analyzed with chi-square or Fisher’s exact tests for cell counts <5. ^2^ Effect sizes were calculated using Pearson’s *r* for continuous variables and Cramér’s V for categorical variables. ^3^ Active celiac disease defined as positive serologies (Tissue Transglutaminase IgA, Deamidated Gliadin Peptide Antibody IgG, Endomysial Antibody IgA), positive endoscopy, or physician-documented concern for active disease within the past 6 months. ^4^ GI symptoms included: abdominal pain, diarrhea, bloating, constipation, nausea, vomiting, increased flatulence, dysphagia, regurgitation, belching, heartburn/reflux, and unspecified bowel changes. ^5^ Non-GI symptoms reported included headaches, brain fog, fatigue, joint pain, rash, and dizziness.

**Table 4:** Logistic regression analysis for predictors of ARFID symptom presence at one-year follow-up (*n* = 126).

	OR (95% CI)	*p*-Value
Age	1.02 (0.98–1.05)	0.29
Sex	0.51 (0.16–1.68)	0.26
Interval weight loss/poor weight gain	18.57 (5.90–66.64)	<0.0001

Note: Age and sex are included as clinically relevant covariates.

## Data Availability

The original contributions presented in this study are included in the article. Further inquiries can be directed to the corresponding author.

## Supplementary Materials

The following supporting information can be downloaded at: https://www.mdpi.com/article/10.3390/nu18101585/s1, Table S1: Characteristics of PEDIATRIC patients by ARFID symptom presence at CeD consultation (*n* = 123); Table S2: Characteristics of ADULT patients by ARFID symptom presence at CeD consultation (*n* = 130).

## Abbreviations

The following abbreviations are used in this manuscript:

ARFID	Avoidant/restrictive food intake disorder
GI	Gastrointestinal
CeD	Celiac disease
NCGS	Non-celiac gluten sensitivity
BMI	Body mass index
GFD	Gluten-free diet
