# Supplementary material for: Avoidant/Restrictive Food Intake Disorder Is Common in Adult and Pediatric Patients with Celiac Disease and Non-Celiac Gluten Sensitivity
Source: Nutrients. 2026 May 16;18(10):1585. doi: 10.3390/nu18101585 (PMC13210292; doi:10.3390/nu18101585)
Supplement: Supplementary file 1 [file nutrients-18-01585-s001.zip › nutrients-4267499-supplementary.pdf]

Supplemental Table S1. Characteristics of PEDIATRIC patients by ARFID symptom presence at CeD consultation (n=123).

Pediatric Consultation	ARFID symptoms (n=28)	No ARFID symptoms (n=95)	p-value[1]	V or d[2]
**Age at consult,** mean (SD) years	10.5 (4.7)	10.8 (4.7)	0.79	0.06
**Sex** – female, n (%)	17 (60.7%)	62 (65.3%)	0.83	0.04
**Race, n (%)**			0.77	0.12
American Indian or Alaskan Native	0 (0%)	0 (0%)		
Asian	0 (0%)	1 (1.1%)		
Black or African American	1 (35.7%)	1 (1.1%)		
Native Hawaiian or Pacific Islander	0 (0%)	0 (0%)		
White	21 (75%)	76 (80%)		
Other	3 (10.7%)	6 (6.3%)		
Unknown	3 (10.7%)	11 (11.6%)		
**Ethnicity, n (%)**			0.26	0.15
Hispanic/Latino(a)	4 (14.3%)	5 (5.3%)		
Not Hispanic/Latino(a)	21 (75%)	81 (85.3%)		
Unknow	3 (10.7%)	9 (9.5%)		
**BMI**				
**BMI percentile,** mean (SD)	35.2 (32.4)	55.5 (29.1)	0.005	0.68
**Biopsy-confirmed celiac disease,** n (%)[3]	19 (67.9%)	73 (76.8%)	0.47	0.09
**Presence of GI Symptoms at consult,** n (%)[4]	28 (100%)	86 (90.5%)	0.2	0.15
**Presence of non-GI symptoms at consult,** n (%)[5]	25 (89.3%)	70 (81.4%)	0.5	0.09
**Comorbidities,** n (%)				
History of anxiety	9 (32.1%)	19 (20.2%)	0.29	0.12
History of depression	2 (7.1%)	9 (9.6%)	0.99	0.04
History of other psychiatric disorder	0 (0%)	6 (6.4%)	0.38	0.12
History of developmental disorder	7 (25%)	10 (10.8%)	0.11	0.17
**On GFD at consultation,** n (%)	16 (57.1%)	44 (46.3%)	0.43	0.09
**On non-GFD exclusion diet at consultation,** n (%)	11(39.2%)	17(18.1%)	0.04	0.21
**History of weight loss/poor weight gain,** n (%)	21 (75%)	38 (40%)	0.002	0.29
**History of poor linear growth,** n (%)	10 (35.7%)	21 (22.1%)	0.23	0.13

Supplemental Table S2. Characteristics of ADULT patients by ARFID symptom presence at CeD consultation (n=130).

Adult Consultation	ARFID symptoms (n=19)	No ARFID symptoms (n=111)	p-value[1]	V or d [2]
**Age at consult,** mean (SD) years	35.6 (16.8)	37.8 (15.4)	0.6	0.14
**Sex** – female, n (%)	14 (73.7%)	93 (83.8%)	0.46	0.09
**Race, n (%)**			0.11	0.24
American Indian or Alaskan Native	1 (5.3%)	0 (0%)		
Asian	0 (0%)	0 (0%)		
Black or African American	0 (0%)	1 (0.9%)		
Native Hawaiian or Pacific Islander	0 (0%)	0 (0%)		
White	17 (89.5)	104 (93.7%)		
Other	0 (0%)	4 (3.6%)		
Unknown	1 (5.3%)	2 (1.8%)		
**Ethnicity, n (%)**			0.44	0.11
Hispanic/Latino(a)	0 (0%)	5 (45%)		
Not Hispanic/Latino(a)	19 (100%)	102 (91.9%)		
Unknown	0 (0%)	4 (3.6%)		
**BMI,** mean (SD)	21.9 (5.17)	25.2 (5.18)	0.02	0.64
**Biopsy-confirmed celiac disease,** n (%)[3]	17 (89.5%)	86 (77.5%)	0.38	0.10
**Presence of GI Symptoms at consult,** n (%)[4]	19 (100%)	106 (95.5%)	0.77	0.08
**Presence of non-GI symptoms at consult,** n (%)[5]	19 (100%)	92 (86.8%)	0.2	0.15
**Comorbidities,** n (%)				
History of anxiety	6 (31.6%)	36 (32.4%)	1	0.01
History of depression	8 (42.1%)	23 (20.7%)	0.08	0.18
History of other psychiatric disorder	3 (15.8%)	6 (5.4%)	0.25	0.14
History of developmental disorder	3 (15.8%)	7 (6.3%)	0.33	0.13
**On GFD at consultation,** n (%)	18 (94.7%)	88 (79.3%)	0.2	0.14
**On non-GFD exclusion diet at consultation,** n (%)	9 (47.4%)	37 (33.3%)	0.36	0.10
**History of weight loss/poor weight gain,** n (%)	13 (68.4%)	39 (35.8%)	0.02	0.24
